# Golden jackal (*Canis aureus*) in the Czech Republic: the first record of a live animal and its long-term persistence in the colonized habitat

**DOI:** 10.3897/zookeys.641.10946

**Published:** 2016-12-16

**Authors:** Klára Pyšková, David Storch, Ivan Horáček, Ondřej Kauzál, Petr Pyšek

**Affiliations:** 1 Department of Ecology, Faculty of Science, Charles University, Viničná 7, CZ-12844 Prague 2, Czech Republic; 2 Institute of Botany, Department of Invasion Ecology, The Czech Academy of Sciences, CZ-25243 Průhonice, Czech Republic; 3 Center for Theoretical Study, Charles University and The Czech Academy of Sciences, Jilská 1, CZ-11000, Prague 1, Czech Republic; 4 Department of Zoology, Faculty of Science, Charles University, Viničná 7, CZ-12844 Prague 2, Czech Republic

**Keywords:** Golden jackal, Habitat, Persistence, Range expansion

## Abstract

A golden jackal (*Canis aureus*) individual was recorded ~40 km east of Prague in the Czech Republic. It is the first record of a living golden jackal in the country; up to now several individuals have been recorded but all of them were either shot dead or killed by a vehicle. The observed animal was documented by camera traps set up for research of carnivore diversity in different habitats in the study area. It was first photographed on 19 June 2015, and in total there were 57 records made by 12 traps until 24 March 2016 when the animal was still present in the area. Forty-nine of the 57 records were made in a shrubby grassland over an area of ~100 ha, 39% of sightings were during the day and 61% in the night. There were two distinct peaks in the circadian activity of the animal, from 4 to 10 a.m., and from 6 p.m. to midnight. We also review the verified records of the golden jackal in the Czech Republic, some of which were only published in local hunting magazines. However, the observation reported in this paper represents the first evidence of a long-term occurrence in Europe of the same golden jackal individual, that persisted for at least nine months and over winter, northwest of Hungarian-Austrian border where the population has been known to reproduce.

## Introduction

Ongoing global change is bringing about shifts in species distributions that include both the spread of populations of invading species and range expansions or contractions of native biota (e.g. Thuiller et al. 2008; Walther et al. 2009; Langham et al. 2015). In Europe this is typically reflected in species moving from the south-eastern part of the continent to the north-west, most often in response to increasing temperatures that allow organisms to colonize areas that were previously unsuitable. Examples cover a wide range of plants and animals (e.g. Walther et al. 2007; Robinet and Roques 2010), including vertebrates. One species that has received much attention because of its range expansion in recent decades is the golden jackal (e.g. Arnold et al. 2012; Šálek et al. 2014; Trouwborst et al. 2015).

The golden jackal (*Canis aureus* L.) is a canid that was traditionally considered native to northern Africa and southern Eurasia, with natural distribution ranging from north of Tanzania in Africa to the Middle-East to Thailand in Asia (Sillero-Zubiri et al. 2004, Jhala and Moehlman 2008). Yet, current genomic analyses (Koepfli et al. 2015) provide a robust evidence of a deep divergence between the Eurasian clade of *Canis aureus* s. str. and the African populations (including those from North Africa) that form a sister clade to *Canis lupus*, which is to be thus referred as a separate species, *Canis anthus*. This work thus confirmed taxonomic separation based on cranial morphological features, suggested first by Spassov (1989).

In Europe, golden jackals naturally occur in the southeastern part of the continent, the Balkan Peninsula (Demeter and Spassov 1993; Krystufek et al. 1997; Stoyanov 2012), but in recent decades the species has started expanding towards the northwest. After undergoing a population decline and local extinctions in the Pannonian region in the mid-20th century, conservation measures and lowered hunting pressure has resulted in the species not only starting to recolonize its former range, but also to spread northwards to regions of Europe where it has never occurred naturally (Krystufek et al. 1997; Lapini et al. 2009; Krofel 2009; Mihelič and Krofel 2012; Trouwborst et al. 2015). During the first decade of the present millennium it became a common and regularly reproducing species in Hungary and East Austria (Szabó et al. 2009) and there have been reports of it occurring as far to the north as Denmark (http://cphpost.dk/news/european-jackal-found-in-denmark.html) and Estonia (http://goldenjackalaround.blogspot.com/2013/03/golden-jackal-survey-in-w-estonia.html), and to the west as Netherlands in 2016 (http://www.wageningenur.nl/nl/Expertises-Dienstverlening/Onderzoeksinstituten/Alterra/show/Eerste-goudjakhals-gezien-in-Nederland.htm). All records documenting the range expansion are very recent (2015, 2013 and 2016, respectively), reported on internet news servers, and refer to dead animals. Overall spread towards the regions in Europe located beyond this species’ historical native range is summarized in the most recent review of the golden jackal historical and current distribution that also deals with the legal status of the species in EU (Trouwborst et al. 2015).

Several factors are assumed to have promoted the dispersal of golden jackals during recent decades. Climate change could play a role by reducing dispersal barriers due to unsuitable climatic conditions in the north of Europe, as suggested for other species (e.g. Walther et al. 2007, 2009). However, the effect of this factor should not be overestimated for a representative of such a highly adaptive guild of carnivores, and a direct evidence of how climate change might have affected this species’ spread is missing. Other suggested factors are human-caused changes in the overall character of landscapes (Šálek et al. 2014), the lack of natural predators, particularly wolves (*Canis lupus*) (Giannatos 2004; Arnold et al. 2012), and also a high degree of ecological tolerance in golden jackal individuals (Banea et al. 2012; Šálek et al. 2014). The golden jackal is omnivorous and can survive in various habitats ranging from arid environments to evergreen forests and it also seems to be able to migrate through high-elevation areas (Sillero-Zubiri 1996). Last but not least, it should be remembered that the observed range expansion is essentially favoured by a pronounced capability of the species for a long-distance leptokurtic disperal. Rutkowski et al. (2015) report a number of records of long-distance movements and demonstrate a dual origin of the population in Baltic region recently established first by immigrants of the Caucasian haplotype followed by those of the south-eastern European origin.

In this paper we (i) report the first occurrence of a living individual of the golden jackal in the Czech Republic, and (ii) provide details on this animal’s persistence in the study area over a period of nine months. To put our observation in a wider geographical context, we (iii) review the available reports on the occurrence of golden jackal in the Czech Republic and the neighbouring countries, with the aim of separating reliable records from those not supported by rigorous evidence.

## Golden jackal in the Czech Republic and neighbouring countries: historical overview

The golden jackal came to the Czech Republic from the south, probably through Austria. The first record in Austria is from Styria in 1987 (Humer 2006) and some of the later observations refer to localities close to the Czech-Austrian border (Hoi-Leitner and Kraus 1989; Bauer and Suchentrunk 1995). In Slovakia, the first record is allegedly from 1947; Feriancová-Masárová and Hanák (1965) report a find of furs of one adult and three young animals in a fur collection point in Bratislava to where they were brought from the Žitný ostrov island on the Danube river; however, the observation remains only anecdotal, unconfirmed by a physical evidence (Hell and Bleho 1995). Later on, an individual was shot near Čierna nad Tisou, eastern Slovakia, in the spring of 1989 (Mošanský 1995) but was at first mistaken for a fox and only later recognized as a golden jackal, documented by an incomplete skull. Another record is a shot animal from the Tríbeč Mountain, February 1995, identified based on skull (see details in Hell and Bleho 1995). At least four other animals were shot between 1989 and 2001 and there were some unconfirmed sightings from central Slovakia in 2008 (Arnold et al. 2012). Concerning arrival to other countries neighbouring the Czech Republic, the golden jackal first appeared in Germany in 1996 (Möckel 2000) and the first record in Poland is from 13 April 2015 – a carcass was found in western Poland near German border. There were a couple more confirmed observations documented by photographs in eastern Poland later that year (Kowalczyk et al. 2015). All of these records refer to occasional observations with no sign of a long-term persistence of the animals in sites where they were spotted. Of the European regions to which golden jackals migrated, they are known to reproduce only in Hungary and Austria with the first evidence of reproduction in the latter country reported in the Austrian-Hungarian border area near the Neusiedler See lake (Arnold et al. 2012, Herzig-Straschil 2007).

The first, albeit unconfirmed, report of the golden jackal’s presence in the Czech Republic is from May of 1998, of two individuals reportedly sighted in central Bohemia near Kropáčova Vrutice, district Mladá Boleslav (Suchomelová 1999). The first confirmed record, however, comes from eight years later (19 March 2006) when a carcass of an adult golden jackal was found by the side of the road near Podolí village, close to town of Uherské Hradiště in Moravia, the eastern part of the country (Koubek and Červený 2007). The next two individuals were also found as a result of road kill, one of them again in Moravia, near Brno, in December 2010 (Forejtek et al. 2011), and the third one in Václavice, central Bohemia, in 2011 (Kadlec 2012). In July 2014 a golden jackal was shot in northern Moravia and later stuffed and put in a museum in Nový Jičín (Hudeček and Jakubec 2014); in the same region, another individual was shot by a hunter near Otice on 16 January 2014 (http://www.nowiny.pl/96628-myslal-ze-strzela-do-psa.html), unfortunately this internet record cannot be considered verified. Beside these, numerous other unconfirmed sightings were made in various parts of the Czech Republic – Anděra (2014) mentions 10 non-verified reports between 2004–2012, all of them but one in the easternmost part of the Czech Republic. Other allegedly new sightings are occasionally reported on internet (e.g., http://www.rozhlas.cz/zpravy/priroda/_zprava/v-lesich-a-na-loukach-v-okoli-sternberka-se-objevili-sakali--1542857). The complete overview of the golden jackal’s verified finds in the Czech Republic is presented in Table 1.

**Figure 1.**
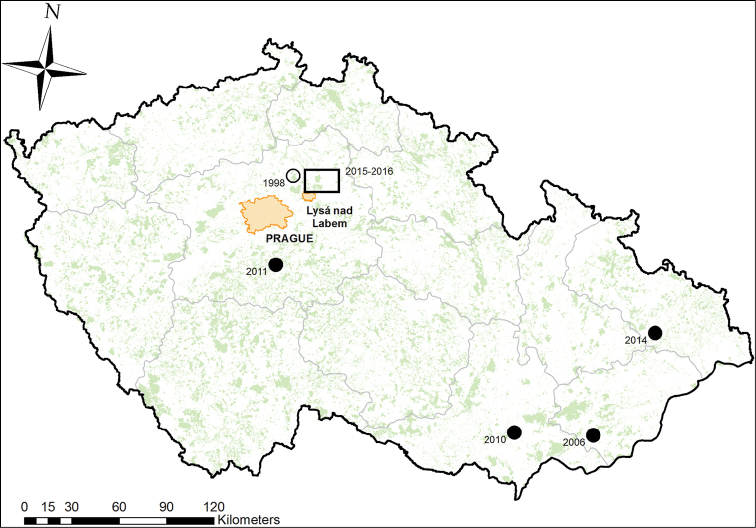
Location of the study area in central Bohemia, western part of the Czech Republic (black rectangle). Previous records relating to dead animals (solid circles), and the first unconfirmed observation (empty circle) are also shown. The records are given by the year of observation, see details in Table 1.

**Table 1.** Overview of verified published records of golden jackal (*Canis aureus*) in the Czech Republic. The records always refer to a single animal. Bohemia is the western and Moravia eastern part of the Czech Republic. See Fig. 1 for location of the finds.

Year	Location	Evidence	Reference
2006 (March)	Podolí (distr. Uherské Hradiště, southern Moravia)	Road kill	Koubek and Červený 2007
2010 (December)	Klobouky u Brna (distr. Brno, southern Moravia)	Road kill	Forejtek et al. 2011
2011 (September)	Václavice^1^ (distr. Benešov, central Bohemia)	Road kill	Kadlec 2012
2014 (July)	Kunín (distr. Nový Jičín, northern Moravia)	Shot	Hudeček and Jakubec 2014
2015–2016	Milovice (distr. Nymburk, central Bohemia)	Photographs	this study

1 This location is erroneously given as Vranovice in Anděra (2014).

## Methods

We used UOVision UV535 Panda camera traps with a motion sensor and infrared night flash that allows for monitoring animals without disturbing them. In May–June 2015 we placed 73 camera traps so as to cover a range of habitats in a relatively untransformed landscape in central Bohemia, ~40 km east of Prague, ~6 km north-east of the town of Lysá nad Labem, near Milovice (Fig. 1). The total size of the area monitored with camera traps is estimated at 709 ha. The photos were being downloaded approximately once a month from June 2015 to March 2016. The focus of the project was to survey carnivores in general, with no primary focus on golden jackal. The results thus represent a random sampling of its presence in the study area over the sampled period.

## Results

The site in which the golden jackal was observed is located approximately 2 km from the nearest village and is surrounded by forests and fields (the average distance from the nearest settlement of the camera traps which recorded the animal was 1.99 km, range 1.60–2.46 km). There is a golf course and an airport nearby. It is not a remote and quiet area and the region is quite densely populated.

**Figure 2.**
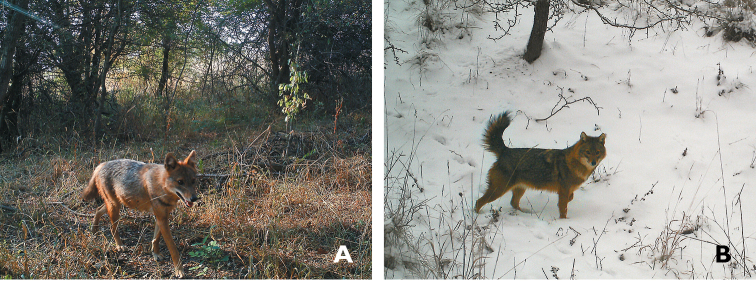
Photographs of a golden jackal (*Canis aureus*) individual in the summer (**A**) and winter (**B**).

The photographs made it possible to determine that the animal was a golden jackal based on morphological characteristics, size and coloration (Fig. 2). We also recorded an individual howling back to the recoding of golden jackal around midnight for about 30 seconds (see Suppl. material 1 for the record). Our determination was verified by Boris Krystufek (personal comm.). It is highly likely that the observed animal was one individual, an adult, the sex of which cannot be determined based on photographs. N. Spassov (in litt.) suggested that in certain respects the observed individual differs from the mean phenotype of the species, namely by its long legs and ears, white spot in the fingers from the front left foot (at a summer photograph; Fig. 2A), and a non-typical winter coloration with the long tail position (Fig. 2B).

**Figure 3.**
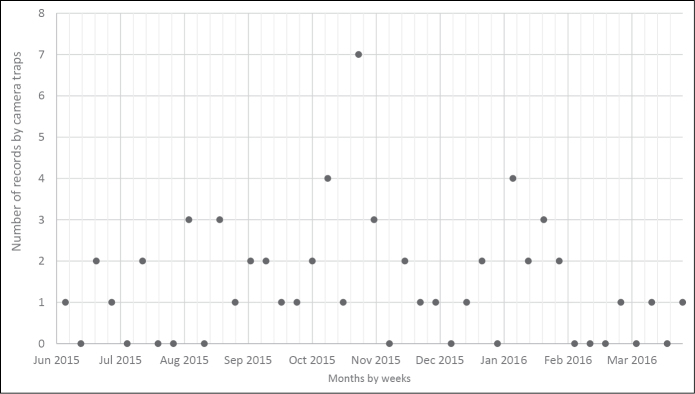
Distribution of the records of golden jackal. The numbers of photographs per week are given.

**Figure 4.**
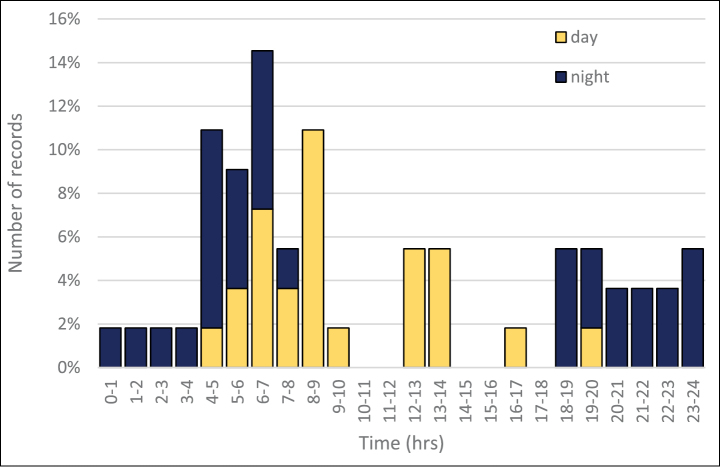
Distribution of the golden jackal (*Canis aureus*) individual’s circadian activity over the study period. Records from the whole monitoring period were pooled and related to the time of the day in which the photographs were taken.

The first photo of a golden jackal’s individual was taken on 19 June 2015 at 8:51 (Fig. 2). Since then the animal was photographed 57 times, by 12 camera traps, with records rather evenly distributed over the sampled period that spanned over 10 months or 40 weeks, until March 2016 (Fig. 3), when the animal was still present in the monitored area. Forty-nine records were made in a shrubby steppe-like grassland with hawthorn (*Crataegus* sp. div.) and blackthorn (*Prunus spinosa*) dominating the shrub layer with varying cover; in some places the cover is quite dense, some parts are more open with grass (Fig. 2). The remaining eight records were in a nearby deciduous forest. The animal was being observed over an area of ~100 ha, with 39% of sightings (22) during the day and 61% (35) in the night. There are two distinct peaks in the circadian activity, at dusk and dawn. The animal was most active between 4 and 7 a.m., with as many as 32% of all records made within these two hours (Fig. 4).

## Discussion

### Systematic sampling reveals long-term persistence: on the way to establishment of golden jackal in the Czech Republic?

Recently, records of the golden jackal individuals have started to be reported from various European regions with increasing frequency, suggesting an ongoing range expansion of this species from the area of its native distribution towards north-west of the continent (Trouwborst et al. 2015). Our observation is the first report of a living animal in the Czech Republic where the four animals previously recorded were road kill or shot. More importantly, it is the only evidence of the long-term persistence of a living animal in the same area available for the region north-west of Hungary and its border with Austria where the species is known to reproduce (Szabó et al. 2009) – earlier records across Europe are mostly vagrants (Trouwborst et al. 2015). Due to systematic random sampling across a large section of the landscape, we were able to prove that the golden jackal individual in our study area, most likely a single individual, occurred on at least ~100 ha area, for eight months so far, and it is still present there at the time of us reporting this find, as of September 2016. In good agreement with literature records, most of activity appeared during night hours, the repeated exploratory behaviour (e.g. controls of badger hole entrance) was recorded also at daytime, surprisingly even around noon.

Although the winter that the animal in our study area survived was rather mild, reports from more northerly located regions of Europe, such as Denmark or Estonia, indicate that this would not be a necessary condition for survival. Moreover, the golden jackal is assumed to be highly adaptive (Šálek et al. 2014) which makes him well suited for establishment in the rather heavily used and densely populated central European landscape.

It is important to note that until now the occurrence in the Czech Republic has been demonstrated by incidental records only, no systematic search was undertaken and the actual distribution of the species is generally unknown. Nevertheless, it is almost certain that the population size of the golden jackal in this country is much higher than previously thought (see Anděra 2014). This, together with the obvious capability for long-term persistence and survival in a suitable habitat as documented in our paper, allows us to predict that the establishment of golden jackal in the Czech Republic in the near future is very probable, and so is the spread of this species further to the north-west. The animal we report here will be further monitored within the ongoing project focused on recording the carnivore diversity in the study area. It remains to be seen whether we will be able to observe the initial phase of establishment. The differences in phenotype of the observed individuals from mean character state of the species evokes a question to which degree a hybridization with domestic dog has been included in the expansion history of the species. Regarding a minute genetic distance between both the species (e.g. Agnarsson et al. 2010) obviously no genetic barrier against hybridization does exist and a lack of social control on alien species mating during a leptokurtic dispersal can be expected as well. Multiple cases of jackal hybridization with domestic dog are well known (e.g. Leonard et al. 2014, Galov et al. 2015).

### Nativity, conservation and legislation: Neither flesh nor red herring?

With the recent expansion of the golden jackal, there has been much discussion about whether or not to treat it as an alien species in countries it recently colonized. Although the recent expansion of the golden jackal in the Baltic countries has generated concerns about its possible negative effects on other wildlife species and livestock via predation or transmission of pathogens, and has led to it being labelled as a potentially invasive alien species, the prevailing opinion in other countries does not support this attitude (Banea et al. 2015; Rutkowski et al. 2015; Science for Environment Policy 2016).

Although some authors argue that invasion is an ecological process, the key feature of which is introduction by humans and subsequent spread, and impact should not be part of the definition (Blackburn et al. 2011), the IUCN definition for an invasive species requires that it has an impact on environment in the new range (IUCN 2000). The golden jackal in the Czech Republic and elsewhere in Europe does not meet the IUCN requirement for an invasive species; all the animals are assumed to have arrived on their own, with intentional release not implicated. Other definitions from the field of invasion biology, based on introduction pathways, do not necessarily require intentional introduction but the process of introduction needs to be related to humans – according to Hulme et al. (2008), a species that arrived without human intervention from an area where it is native should be treated as native. The available evidence is therefore in favour of considering the golden jackal as a species that naturally expands its distribution, making use of the changing conditions.

The latter statement indicates that the conclusion about the golden jackal’s nativity (which implies legal protection which has become an issue recently; Science for Environment Policy 2016) has a flip side – if it is not alien to central Europe, is it native? This begs the question to what extent the expansion is natural? The factors driving or facilitating the golden jackal’s expansion are still not entirely clear although land use changes, as well as climate change, are most likely implicated (Giannatos 2004; Arnold et al. 2012; Šálek et al. 2014). Moreover, it is also suggested that the expansion might be easier in current landscape where wolves, natural intra-guild predators of golden jackals, are uncommon or absent (Giannatos 2004; Arnold et al. 2012). However, this argument is rather speculative because in central European temperate landscapes the two species differ in their habitat preferences. This allows for quasi-supported speculation that had it not been for the indirect effect of humans, the golden jackal might not have reached that far to the northwest of Europe where it is currently present. It is not the primary aim of this article to deal with this issue that has been recently thoroughly reviewed in Trouwborst et al. (2015) but we think it is worth pointing out that opinions about this species’ status in Europe may continue to differ region by region in the future, with good reasons on both sides.

## Supplementary materials

Supplementary material 1 Goledn jackal’s howling record Data type: multimedia Explanation note: Recording of an individual howling back to the recording of golden jackal. Recorded on 15 September 2015 around midnight. This dataset is made available under the Open Database License (http://opendatacommons.org/licenses/odbl/1.0/). The Open Database License (ODbL) is a license agreement intended to allow users to freely share, modify, and use this Dataset while maintaining this same freedom for others, provided that the original source and author(s) are credited.Klára Pyšková, David Storch, Ivan Horáček, Ondřej Kauzál, Petr Pyšek
