# Supplementary figures and images for: Golden jackal (Canis aureus) in the Czech Republic: the first record of a live animal and its long-term persistence in the colonized habitat
Source: Zookeys. 2016 Dec 16;(641):151–63. doi: 10.3897/zookeys.641.10946 (PMC5240352; doi:10.3897/zookeys.641.10946)

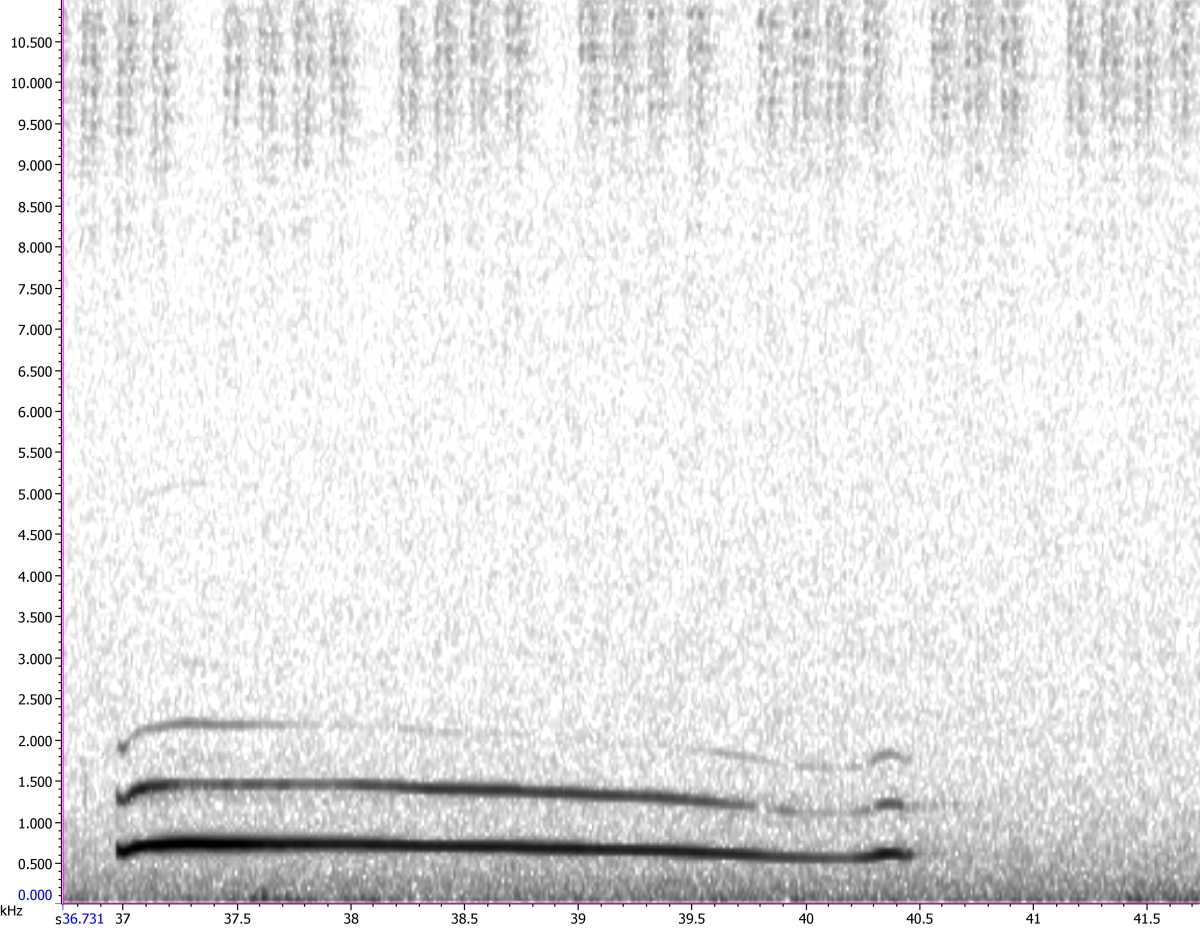

Supplement: Supplementary material 1 — Goledn jackal’s howling record [file zookeys-641-151-s001.png]
